# A comparison of DP-TOF Mass Spectroscopy (MS) and Next Generation Sequencing (NGS) methods for detecting molecular mutations in thyroid nodules fine needle aspiration biopsies

**DOI:** 10.3389/fendo.2022.928788

**Published:** 2022-08-04

**Authors:** Xiao-qin Qian, Enock Adjei Agyekum, Ling-ling Zhao, Run-liu Yu, Xiu-ying Li, De-jian Gu, Na Yan, Ming Xu, Yuan Yuan, Yu-guo Wang, Wu Xin-ping, Fei-ju Xu

**Affiliations:** ^1^ Department of Ultrasound, Affiliated People’s Hospital of Jiangsu University, Zhenjiang, China; ^2^ School of Medicine, Jiangsu University, Zhenjiang, China; ^3^ Department of Ultrasound, Jiangsu Hospital of Integrated Traditional Chinese and Western Medicine, Nanjing, China; ^4^ Research and Development Center, Hangzhou D.A. Medical Laboratory, Hangzhou, China; ^5^ Nanjing D.A. Medical Laboratory, Nanjing, China; ^6^ Key Laboratory of Digital Technology in Medical Diagnostics of Zhejiang Province, Dian Diagnostics Group Co., Ltd., Hangzhou, China; ^7^ Department of Medicine, Zhejiang Digena Diagnosis Technology CO., LTD, Zhejiang, China; ^8^ Department of Ultrasound, The Second Affiliated Hospital of Nanjing Medical University, Nanjing, China

**Keywords:** thyroid nodules, *BRAF gene*, next generation sequencing, mass spectroscopy, fine needle aspiration

## Abstract

Mutations in the B-Raf proto-oncogene, serine/threonine kinase (BRAF), have been linked to a variety of solid tumors such as papillary thyroid carcinoma. The purpose of this study was to compare the DP-TOF, a DNA mass spectroscopy (MS) platform, and next-generation sequencing (NGS) methods for detecting multiple-gene mutations (including BRAF^V600E^) in thyroid nodule fine-needle aspiration fluid. In this study, we collected samples from 93 patients who had previously undergone NGS detection and had sufficient DNA samples remaining. The MS method was used to detect multiple-gene mutations (including BRAF^V600E^) in DNA remaining samples. NGS detection method was used as the standard. The MS method’s overall sensitivity, specificity, positive predictive value (PPV), and negative predictive value (NPV) were 95.8%, 100%, 100%, and 88%, respectively in BRAF^V600E^ gene mutation detection. With a kappa-value of 0.92 (95%CI 0.82–0.99), the level of agreement between these methods was incredibly high. Furthermore, when compared to NGS in multiple-gene detection, the MS method demonstrated higher sensitivity and specificity, 82.9% and 100%, respectively. In addition, we collected the postoperative pathological findings of 50 patients. When the postoperative pathological findings were used as the standard, the MS method demonstrated higher sensitivity and specificity, at 80% and 80%, respectively. Our findings show that the MS method can be used as an inexpensive, accurate, and dependable initial screening method to detect genes mutations and as an adjunct to clinical diagnosis.

## Introduction

Papillary thyroid carcinoma is the most common thyroid cancer of the endocrine system, with a relatively slow progression and a high survival rate (1, 2). Papillary thyroid cancer has been on the rise for three decades (3, 4). Fine-needle aspiration (FNA) cytology is the most commonly used method for diagnosing and categorizing thyroid carcinoma (1). Tumor cells in FNA biopsy samples vary in quantity, quality, and purity, making identification and diagnosis difficult (1). BRAF^V600E^ mutation has been established as an important molecular marker for papillary thyroid cancer diagnosis over the last decade, with a frequency of 65–80% (5, 6). As a result, a sensitive and accurate detection method for the BRAF^V600E^ mutation will aid in the early diagnosis of papillary thyroid carcinoma (7). For the detection of BRAF^V600E^ mutations, amplification-refractory mutation system (ARMS) and next-generation sequencing (NGS) are currently used, particularly NGS, which is a sensitive method in FNA samples with few mutant cells. However, NGS is expensive and inappropriate for the initial screening of all clinical patients. As a result, a more sensitive, low-cost, and accurate detection method is required.

The DNA mass spectroscopy (MS) method is a multiplexed medium-throughput ultra-sensitive mutation detection system. This method has been used successfully to detect mutations in patients with solid tumors, with a reported limit-of-detection frequency of 0.5% (8). It is far more sensitive and specific than other clinical methods currently in use. However, the detection of BRAF ^V600E^ mutations using MS has not been investigated in papillary thyroid carcinoma FNA samples. In this study, we used MS to detect the BRAF^V600E^ mutation in thyroid nodule FNA samples and compared its performance to that of NGS. Our study demonstrates the clinical significance of MS in the early detection of thyroid carcinoma.

## Materials and methods

### Subjects and study design

**Figure 1:**
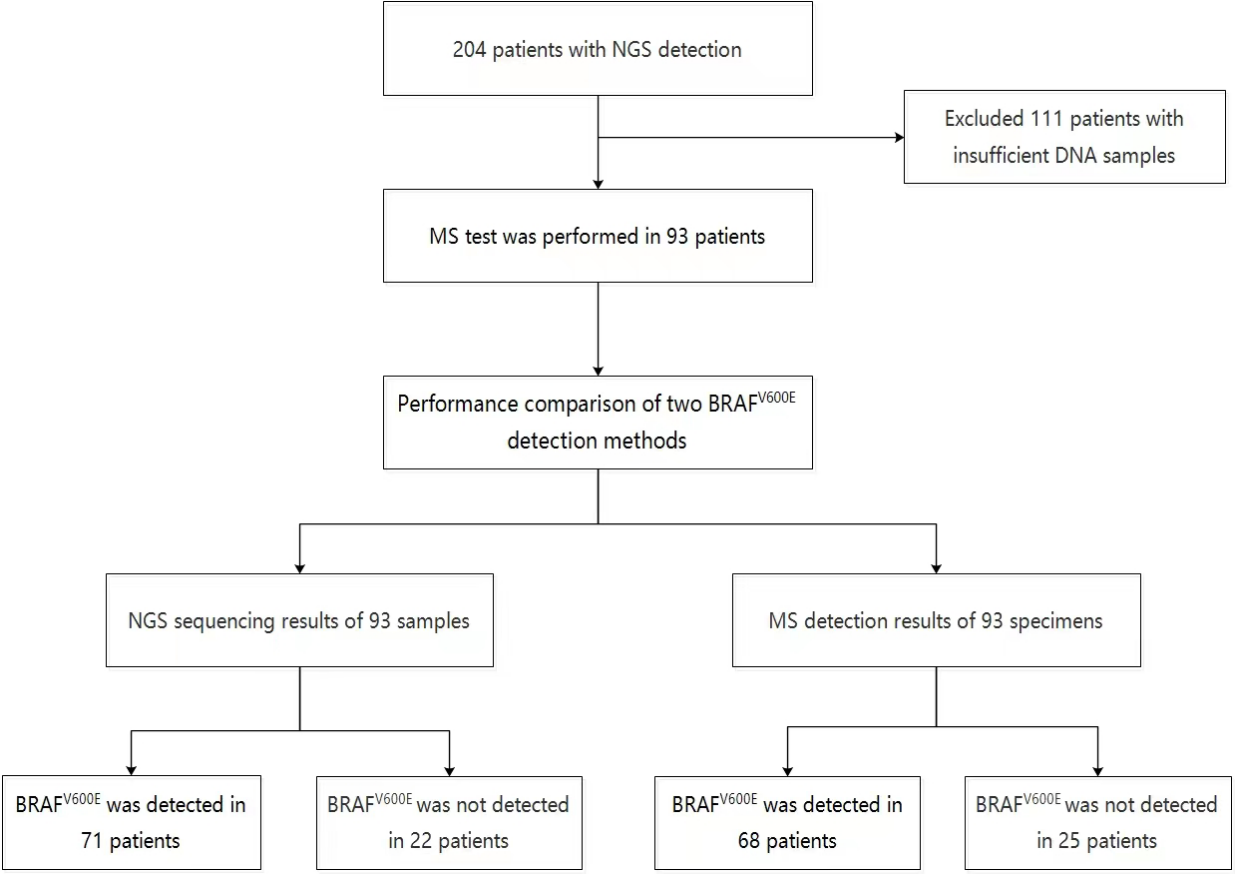
Flow chart of the study.

From January 2020 to January 2022, 204 patients with thyroid nodules who underwent thyroid ultrasound examination and next-generation sequencing (NGS) at Jiangsu Hospital of Integrated Traditional Chinese and Western Medicine and Jiangsu University Affiliated People’s Hospital were analyzed retrospectively. The study then enrolled 93 patients who still had enough DNA samples remaining after next-generation sequencing (NGS) (Fig 1). This study was approved by the Ethics Committee of the two hospitals, and all patients provided written informed consent.

### DNA extraction

The QIAamp DNA Mini Kit (QIAGEN, Germany) was used to extract genomic DNA from thyroid FNA samples, and DNA concentrations were measured using the Qubit (Thermo Fisher Scientific, Waltham, USA).

### DNA sequencing by NGS and MS

A custom-designed NGS panel containing 11 cancer-associated genes, including BRAF^V600E^, KRAS, NRAS, HRAS, TERT, TP53, RET, NTRK1, NTRK3, PAX8, and THADA, was used to perform comprehensive genomic profiling. TruSeq DNA Library Preparation Kit protocols were used to create genomic DNA sequencing libraries. DNA sequencing was carried out on an Illumina CN 500 sequencing system (San Diego, CA). BWA (a Burrows-Wheeler aligner) (9) was used to align the reads to the human genome build GRCh37. MuTect2 (3.4-46-gbc02625) (10) was used to identify single nucleotide variants (SNVs), while GATK was used to identify small insertions and deletions (SIDs) (Indels). The integrative genomics viewer browser was used to validate all final candidate variants.

We created an MS panel with four gene assays: BRAF^V600E^, TERT, TP53, and RET. All four genes are common in papillary thyroid carcinoma (PTC). BRAF^V600E^ and RET were important molecular markers of PTC (1, 11, 12). TERT and TP53 were found to be associated with high aggressiveness (12, 13). The remaining DNA samples from NGS sequencing were used for MS detection. The DNA concentrations were determined using Qubit 3.0. (Thermo Fisher Scientific, Waltham, USA). The production was then carried out at Zhejiang Digena R&D Center, on a high-throughput DP-TOF MassARRAY platform with data analyzed using Typer 4.0 and plate manager 1.0 software.

### Statistical analyses

SPSS 22.0 (SPSS Inc., USA) was used to perform all statistical analyses. The inter-rater agreement (kappa-value) test was used to assess the degree of agreement between the MS and NGS methods. A p-value <0.05 was considered statistically significant.

## Results

### Comparison of NGS and MS for detection of BRAF ^V600E^ mutation in FNA cytology biopsy samples

To compare the efficacy of NGS and MS in detecting BRAF^V600E^ mutation in FNA, samples from patients who had previously undergone NGS detection were collected, and 93 patients with sufficient DNA samples remaining were enrolled in the study, the 93 samples were also detected by MS in this study. In the NGS analysis of 93 patients, 71 were found to have BRAF^V600E^ mutation (69 with only BRAF^V600E^, one with KRAS and BRAF^V600E^, and one with TERT and BRAF^V600E^), 11 with other mutations including KRAS, NRAS, HRAS, and NTRK3, and 11 without gene mutation. In the MS detection of these patients, 68 were found to have BRAF^V600E^ and no gene mutation was detected in 25 cases (**Figure 1**).

The comparative analysis of NGS and MS for the Detection of Molecular Mutations was performed using the BRAF^V600E^ mutation status established from NGS detection as the standard. The MS method’s overall sensitivity, specificity, positive predictive value (PPV), and negative predictive value (NPV) were 95.8%, 100%, 100%, and 88%, respectively (**Tables 1**, **2**). Furthermore, with a kappa-value of 0.92 (95% confidence interval (CI) 0.82–0.99, p<0.001), the level of agreement between the two methods was very high. The two methods had a 96.8% (90/93) coincidence rate, with three patients missed in MS methods (**Table 2**). According to NGS, the frequency of BRAF^V600E^ in the three patients was 1.48%, 0.88%, and 0.75%, respectively. The reason for tracing was that the amount of remaining DNA was insufficient, resulting in insufficient initial abundance and a negative MS test.

**Table 1:** Comparison of NGS and MS methods in detecting *BRAF* V600E mutation from FNA biopsy.

MS vs NGS		
	NGS	
MS	Positive	Negative
Positive	68	0
Negative	3	22
Sensitivity: 95.8%	Specificity: 100%	PPV:100% NPV: 88%

BRAF results of NGS detecting was used as the standard reference. PPV, positive predictive value; NPV, negative predictive value; MS, mass spectroscopy; NGS, next generation sequencing.

**Table 2:** The gene mutations of patients who was negative in MS detected by NGS.

Patient ID	Gene	HGVS	Frequency	MS detecting results	clinical diagnosis
P8	*HRAS*	p.Q61R	19.52%	ND	NA
P30	*NRAS*	p.Q61R	42.99%	ND	Benign
P31	*ETV6-NTRK3*	fusion	NA	ND	Malignant
P32	*HRAS*	p.Q61R	31.66%	ND	Benign
P38	*NRAS*	p.Q61K	26.98%	ND	Malignant
P44	*NRAS*	p.Q61R	1.04%	ND	NA
P47	*ETV6-NTRK3*	fusion	NA	ND	Benign
P48	*CCDC6-RET*	fusion	NA	ND	NA
P57	*KRAS*	p.Q61R	36.12%	ND	NA
P60	*NRAS*	p.Q61R	44.55%	ND	Benign
P62	*BRAF*	p.V600E	0.75%	ND	NA
P78	*BRAF*	p.V600E	0.88%	ND	Malignant
P86	*ETV6-NTRK3*	fusion	NA	ND	Malignant
P91	*BRAF*	p.V600E	1.48%	ND	NA

MS, mass spectroscopy; NGS, next-generation sequencing; NA, not applicable; ND, not detected; HGVS, Human Genome Variation Society.

### Comparison of NGS and MS for detection of multiple gene mutations in FNA cytology biopsy samples

The MS panel in this study examined mutations in four genes, including TP53, TERT, and RET, in addition to BRAF^V600E^. The NGS panel examined 11 different genes for mutations. The results of NGS and MS were compared in order to compare their efficacy in detecting multiple gene mutations. The two methods had an 84.9% (79/93) coincidence rate. The sensitivity, specificity, PPV, and NPV of the MS method were 82.9%, 100%, 100%, and 44%, respectively, as shown in **Table 3**. The level of agreement between the two methods was moderate, with a kappa-value of 0.54 (95% CI 0.34–0.73, p<0.001).

**Table 3:** Comparison of NGS and MS methods in detecting multiple gene mutations from FNA biopsy.

MS vs NGS		
	NGS	
MS	Positive	Negative
Positive	68	0
Negative	14	11
Sensitivity: 82.9%	Specificity: 100%	PPV:100% NPV: 44%

Results of NGS detecting were used as the standard reference. PPV, positive predictive value; NPV, negative predictive value; MS, mass spectroscopy; NGS, next-generation sequencing; Positive, Gene mutations were detected in NGS/MS panel.

In comparison to the NGS method, there were no false positives reported with the MS method. However, due to panel limitations, 11 patients with positive genes outside of the MS panel were found to be negative (**Table 3**). In these 11 patients, two HRAS (all p.Q61R) mutations were found, one KRAS (p.Q61R) mutation, four NRAS (3 p.Q61R and 1 p.Q61K) mutations, three ETV6-NTRK3 fusions, and one CCDC6-RET fusion (**Table 3**). Furthermore, two of the 93 patients had more than one gene mutation (one with KRAS and BRAF, the other with TERT and BRAF^V600E^). MS methods were also used to detect patients who had BRAF^V600E^ and TERT mutations. This result demonstrated that the MS method was capable of detecting multiple genes.

### Relationship between the NGS or MS detection results and postoperative pathological findings

Furthermore, we collected the postoperative pathological findings of 50 patients, 45 of whom were papillary thyroid carcinoma (PTC). We also investigated the relationship between postoperative pathological findings and NGS or MS detection results, using pathological findings as the gold standard. Considering that the genes contained in NGS/MS panel are common genes for thyroid cancer diagnosis and prognosis, positive was defined as the detection of mutations in the NGS or MS panel. The sensitivity, specificity, PPV, and NPV of the MS method were 80%, 80%, 97.3%, and 30.8%, respectively, while the sensitivity, specificity, PPV, and NPV of the NGS method were 88.9%, 0%, 88.9%, and 0%. The level of agreement between clinical diagnosis and MS or NGS was lower, with kappa-values of 0.351 (95% CI 0.06–0.643, p=0.004) and 0.111 (95% CI 0.04–0.179, p=0.4), respectively.

Both MS and NGS showed higher sensitivity, as evidenced by the above results. However, some patients were missed in both methods. In the MS analysis, 9 patients were found to have thyroid cancer despite having a negative MS test result. NGS detected gene mutations in four patients out of nine, including one with BRAF^V600E^, one with NRAS, and two with NTRK3 fusion. Gene mutations, however, were found in all five patients with clinically benign nodules, with one having the BRAF^V600E^ mutation (**Table 4**). These findings suggest that multigene testing could be used as an adjunct to clinical diagnosis, but it must be used in conjunction with other clinical methods.

**Table 4:** Relationship between the NGS or MS detection results and clinical diagnosis.

MS vs clinical diagnosis		
	clinical diagnosis	
MS	Malignant	Benign
Positive	36	1
Negative	9	4
Sensitivity: 80%	Specificity: 80%	PPV:97.3% NPV: 30.8%
**NGS vs clinical diagnosis**		
	**clinical diagnosis**	
**NGS**	**Malignant**	**Benign**
Positive	40	5
Negative	5	0
Sensitivity: 88.9%	Specificity: 0	PPV:88.9% NPV: 0

Results of clinical diagnosis were used as the standard reference. PPV, positive predictive value; NPV, negative predictive value; MS, mass spectroscopy; NGS, next-generation sequencing; Positive, Gene mutations were detected in NGS/MS panel.

## Discussion

We demonstrated in this study that both NGS and MS are effective methods for detecting BRAF ^V600E^ mutations in FNA biopsy samples of patients with thyroid nodules, with a strong inter-rater agreement, high specificity, and high PPV. Furthermore, the MS method demonstrated significant potential as a screening method for gene mutation detection and as an adjunct to postoperative pathological findings.

Several next-generation sequencing studies, including whole-genome sequencing (14), whole-exome sequencing (15, 16), and targeted sequencing (17, 18) have recently been conducted to investigate the genetic changes in papillary thyroid carcinoma. B-Raf proto-oncogene, serine/threonine kinase (BRAF^V600E^), and telomerase reverse transcriptase (TERT) promoter mutations were the most frequently identified in papillary thyroid carcinoma. According to Cancer Genome Atlas (TCGA) cohort and several studies, the most common alterations are BRAF^V600E^ mutation in 62% (19, 20), TERT mutation in 22% (21), and RAS (including HRAS, NRAS, and KRAS) mutation in 13% (19). A high prevalence of BRAF^V600E^ mutations was also found in papillary thyroid carcinoma patients in a Chinese cohort study (22). In patients with papillary thyroid carcinoma, Khan and colleagues discovered that 94 percent of BRAF mutations were BRAF ^V600E^ mutations (21). Both the National Comprehensive Cancer Network (NCCN) and the Chinese Society of Clinical Oncology (CSCO) recommend testing for BRAF^V600E^ mutations as a supplement to clinical diagnosis. As a result, preliminary BRAF screening is required for all patients with thyroid nodules to aid clinical diagnosis. The BRAF^V600E^ mutation was found in 76.3% of the patients in this study, which was consistent with a Chinese cohort study (23).

TERT in 1.1%, and RAS (including HRAS, NRAS, and KRAS) mutation in 8.6% of the patients in this study.

Because of its high throughput, multi-gene coverage, and high precision, next-generation sequencing (NGS) is a widely known method for detecting solid tumor mutations. However, due to the high throughput and high cost of the equipment, NGS is not suitable for all thyroid tumors or nodules patients for initial screening, particularly for the detection of a BRAF gene (24). The MS method is a multiplexed ultrasensitive mutation detection system with a medium throughput. This method had previously been used successfully to detect mutations in patients with solid tumors, with a reported limit-of-detection frequency of 0.1% (8). In this study, we compared the efficacy of MS and NGS methods for detecting BRAF mutations. Our findings show that the MS method is a reliable and sensitive method for detecting BRAF^V600E^ mutations in thyroid tumors or nodules in patients’ FNA biopsy samples. Compared with NGS detection, the MS demonstrated greater sensitivity and specificity. However, the MS method did not detect three patients with BRAF^V600E^. False-negative results were complicated because the study used retrospective samples. According to the NGS results, the BRAF mutation frequency in these three patients was relatively low. We hypothesized that the false-negative detection was a lack of sufficient tumor DNA in the remaining samples. The absence of false-positive cases demonstrates the MS method’s potential in clinical applications.

Furthermore, we compared the efficiency of MS and NGS methods in detecting multiple-gene mutations. The MS method maintained high specificity and sensitivity, as expected. However, 11.8% of patients were reported negative because they had mutations that were not found in the MS panel. Intriguingly, MS results from NGS were consistent in a patient with BRAF^V600E^ and TERT mutations. Previous research has shown that patients with both BRAF^V600E^ and TERT mutations have a poor prognosis, so simultaneous multigene screening is necessary (13).

The MS and NGS results were also evaluated using the postoperative pathological findings as the standard. The MS method had higher sensitivity and specificity with pathological findings, and the majority of false-negative patients had out-of-panel mutations. Although the 11-gene panel of NGS demonstrated higher sensitivity due to the greater number of non-BRAF mutations covered, NGS demonstrated lower specificity due to non-BRAF mutations detected in benign lesions (12, 25). Three of the five patients with benign lesions had RAS mutations, and one had an NTRK3 fusion. Previous research has shown that these mutations can be found in both malignant and benign lesions (11, 12, 25). In addition, all malignant in our study were PTC. Previous studies have confirmed that non-BRAF genes were more common in other subtype thyroid carcinoma patients, which may have higher diagnostic value. For example, RAS mutation in follicular thyroid carcinoma was more common than BRAF (12). Moreover, 7 patients had the results of clinical diagnosis in 11 patients with non-BRAF mutations. Of the 7 patients, 4 (57.14%) patients were benign lesions, and 3 of 4 patients harbored RAS mutations. Although several studies have demonstrated that multigene testing can improve the specificity of clinically assisted diagnosis (26–28). Previous research and CSCO guidelines have shown that RAS mutations have an unsatisfactory clinical impact on the management of thyroid nodules (29). As a result, the NCCN and CSCO guidelines recommend BRAF as the primary screening gene for adjunctive diagnosis, and these non-BRAF genes are not required for initial screening. These findings imply that the MS method can be used as a primary screening method for molecular detection in patients.

There were a few limitations to this study as well. Due to the small number of patients in our cohort who had TERT and TP53 mutations, the feasibility of the MS method in this population needs to be investigated further. Second, the importance of multiple-gene detection should be discussed further. Because our study was retrospective, residual DNA from NGS detection was used as samples in MS detection, limiting the sample size of this study. The possibility of full-process detection of clinical FNA samples should be investigated further.

## Conclusion

Finally, our findings showed that the MS method was a precise and dependable alternative for detecting BRAF mutations in patients with thyroid nodules. Furthermore, the MS method as primary screening for molecular detection was promising.

## Data availability statement

The raw data supporting the conclusions of this article will be made available by the authors, without undue reservation.

## Ethics statement

The studies involving human participants were reviewed and approved by Ethics Committee of Jiangsu Hospital of Integrated Traditional Chinese and Western Medicine and Jiangsu University Affiliated People’s Hospital. All procedures were in accordance with the 1964 Declaration of Helsinki and its later amendments. The patients/participants provided their written informed consent to participate in this study.

## Author contributions

X-QQ and EA contributed to the conception and design of the study. Y-GW and F-JX contributed to the acquisition of data. L-LZ, R-LY, and X-YL assisted in sample management and experiment operation. F-JX, NY, MX, and D-JG organized the database. NY, F-JX, YY and MX contributed to the confirmation of the authenticity of the data. Y-GW, X-QQ, EA, WX-P and D-JG performed data analysis and interpretation. EA performed review and editing. All authors contributed to the manuscript and read and approved the final manuscript. All authors contributed to the article and approved the submitted version.

## Funding

This study was financially supported by National Natural Science Foundation of China (Project No. 81971629) and Zhenjiang Commission of Science and Technology (Project No. SH2020046).

## Conflict of interests

NY was employed by Dian Diagnostics Group Co., Ltd and MX was employed by Zhejiang Digena Diagnosis Technology Co. Ltd.

The remaining authors declare that the research was conducted in the absence of any commercial or financial relationships that could be construed as a potential conflict of interest.

## Publisher’s note

All claims expressed in this article are solely those of the authors and do not necessarily represent those of their affiliated organizations, or those of the publisher, the editors and the reviewers. Any product that may be evaluated in this article, or claim that may be made by its manufacturer, is not guaranteed or endorsed by the publisher.
